# Adjuvant Radiation in Older Patients With Glioblastoma: A Retrospective Single Institution Analysis

**DOI:** 10.3389/fonc.2021.631618

**Published:** 2021-02-25

**Authors:** Jessica W. Lee, John P. Kirkpatrick, Frances McSherry, James E. Herndon, Eric S. Lipp, Annick Desjardins, Dina M. Randazzo, Henry S. Friedman, David M. Ashley, Katherine B. Peters, Margaret O. Johnson

**Affiliations:** ^1^ Department of Radiation Oncology, Duke University School of Medicine, Durham, NC, United States; ^2^ Department of Biostatistics & Bioinformatics, Duke University School of Medicine, Durham, NC, United States; ^3^ Department of Neurosurgery, The Preston Robert Tisch Brain Tumor Center, Duke University Medical Center, Durham, NC, United States

**Keywords:** glioblastoma, frail elderly, aged, radiotherapy, radiation dose hypofractionation, radiation oncology

## Abstract

**Objectives:**

Standard 6-week and hypofractionated 3-week courses of adjuvant radiation therapy (RT) are both options for older patients with glioblastoma (GBM), but deciding the optimal regimen can be challenging. This analysis explores clinical factors associated with selection of RT course, completion of RT, and outcomes following RT.

**Materials and Methods:**

This IRB-approved retrospective analysis identified patients ≥70 years old with GBM who initiated adjuvant RT at our institution between 2004 and 2016. We identified factors associated with standard or hypofractionated RT using the Cochran-Armitage trend test, estimated time-to-event endpoints using the Kaplan-Meier method, and found predictors of overall survival (OS) using Cox proportional hazards models.

**Results:**

Sixty-two patients with a median age of 74 (range 70–90) initiated adjuvant RT, with 43 (69%) receiving standard RT and 19 (31%) receiving hypofractionated RT. Selection of short-course RT was associated with older age (p = 0.04) and poor KPS (p = 0.03). Eight (13%) patients did not complete RT, primarily for hospice care due to worsening symptoms. After a median follow-up of 37 months, median OS was 12.3 months (95% CI 9.0–15.1). Increased age (p < 0.05), poor KPS (p < 0.0001), lack of MGMT methylation (p < 0.05), and lack of RT completion (p < 0.0001) were associated with worse OS on multivariate analysis. In this small cohort, GTV size and receipt of standard or hypofractionated RT were not associated with OS.

**Conclusions:**

In this cohort of older patients with GBM, age and KPS was associated with selection of short-course or standard RT. These regimens had similar OS, though a subset of patients experienced worsening symptoms during RT and discontinued treatment. Further investigation into predictors of RT completion and survival may help guide adjuvant therapies and supportive care for older patients.

## Introduction

Glioblastoma (GBM) is a malignancy of older adults. The median age at diagnosis is 65 years old, and the incidence increases with age, peaking in the 75–84 years old age group (1). The Stupp trial established the current standard treatment of maximal safe resection followed by adjuvant radiation therapy (RT) for 6 weeks with concurrent and adjuvant temozolomide (2). However, this trial excluded patients >70 years old, and as age is both a negative prognostic factor and predictor of response to RT, other randomized studies have investigated radiation or temozolomide alone for older adults (3–5). The Canadian trial found that in patients ≥60 years old, 40 Gy in 15 fractions was non-inferior to 60 Gy in 30 fractions, with median survival of 5.1 and 5.6 months, respectively (6). The Nordic trial found that in patients >70 years old, 34 Gy in 10 fractions or temozolomide alone both had improved survival compared to 60 Gy in 30 fractions, though the latter group had more patients discontinue treatment (7). NOA-08 found that in patients >65 years old, temozolomide was non-inferior to 60 Gy in 30 fractions (8). In both the Nordic and NOA-08 trials, O^6^-methylguanine DNA methyltransferase (MGMT) promoter methylation predicted a survival benefit from temozolomide (7, 8). More recently, a randomized study of patients ≥65 years old found that addition of temozolomide to the 40 Gy regimen did improve survival from 7.6 to 9.3 months (9).

Based on the above studies, temozolomide with standard or hypofractionated RT are both options for patients >70 years old with good performance status (10). The optimal RT regimen is not clear, though individualized treatment decisions may take into account factors such as age, performance status, and MGMT methylation (11). Standardized geriatric assessments have also been proposed to help guide treatment decisions (12). Overall, utilization of hypofractionated RT in the United States remains low. In several National Cancer Database (NCDB) analyses of older patients with GBM receiving adjuvant RT, only 2.5–20% received a hypofractionated regimen (13–17).

Here, we report our institutional experience with older patients initiating adjuvant RT, focusing on factors affecting the selection of standard or hypofractionated regimens and clinical outcomes.

## Materials and Methods

Following institutional review board approval, we identified patients with GBM who were ≥70 years old at time of pathologic diagnosis and initiated adjuvant RT in our radiation oncology department between 2004 and 2016.

Patient characteristics including age, sex, Karnofsky performance status (KPS), and MGMT methylation status were obtained from the medical record. KPS was documented following maximal resection at the time of radiation oncology consultation. Treatment details including radiation technique, gross tumor volume (GTV), planning target volume (PTV), and receipt of concurrent temozolomide or bevacizumab were also obtained. Standard radiation therapy to primary and boost volumes was delivered per Radiation Therapy Oncology Group (RTOG) guidelines (18, 19). Specifically, the primary PTV consisted of the pre-operative T2-hyperintense GTV plus a 2 cm margin and received 45–50.4 Gy at 1.8–2 Gy/fraction. The boost PTV was the post-operative T1 contrast-enhancing GTV plus a 1.5 cm margin and received a total dose of 59.4–60 Gy at 1.8–2 Gy/fraction. For hypofractionated radiation therapy, the PTV comprised of T1 contrast-enhancing GTV plus a 1.5 cm margin and received 40.05 Gy in 2.67 Gy/fraction. As we used frequent image guidance and stereotactic radiosurgery-capable, custom-molded head immobilization, there was no further expansion for set-up error. PTVs were trimmed where they extended across anatomic boundaries such as the falx, into non-target tissues such as the orbits or outer table of the skull or the scalp. Boost PTVs were also trimmed where they extended into critical organs at risk such as the brainstem and anterior visual pathways. Temozolomide was administered to all patients where possible and dosed per the Stupp trial, and bevacizumab was administered at the discretion of the treating oncologist (2, 20, 21).

### Statistics

Association between clinical characteristics and selection of standard or hypofractionated RT was assessed using the Cochran-Armitage test for trend. Overall survival, measured from date of pathologic diagnosis, was estimated by the Kaplan-Meier method and compared *via* log-rank test. Clinical factors associated with overall survival were evaluated using Cox proportional hazards models. Significance was assumed if p < 0.05. Statistical analyses were performed using SAS 9.4.

## Results

Between 2004 and 2016, 62 older patients initiated adjuvant RT. Baseline patient characteristics are shown in **Table 1**. Overall, patients had a median age of 74 years old, and 34 (55%) were male. Most patients received a resection; 33 (53%) had a gross total resection (GTR) and 10 (16%) had a subtotal resection, while 19 (31%) underwent biopsy only. Forty-four (71%) patients had a Karnofsky performance status (KPS) of ≥70 prior to starting adjuvant RT. MGMT methylation status was known for 46 (74%) patients, and 20 (32%) had MGMT methylation. In patients receiving standard RT, the median initial GTV was 98 cm^3^ and the median boost GTV was 31 cm^3^. In patients receiving hypofractionated RT, the median GTV was 27 cm^3^. Fifty-eight (94%) and 20 (32%) patients received concurrent temozolomide and bevacizumab, respectively.

**Table 1:** Baseline demographics and treatment details.

Characteristic		Standard (N = 43)	Hypofractionated (N = 19)	All (N = 62)
**Age (years)**		74 (70–88)	77 (71–91)	74 (70–91)
**Sex**	Female	17 (40)	11 (58)	28 (45)
	Male	26 (61)	8 (42)	34 (55)
**Maximal resection**	GTR	26 (61)	7 (37)	33 (53)
	STR	7 (16)	3 (16)	10 (16)
	Biopsy only	10 (23)	9 (47)	19 (31)
**KPS**	≥70	32 (74)	12 (63)	44 (71)
	<70	6 (14)	4 (22)	10 (16)
	Unknown	5 (12)	3 (16)	8 (13)
**MGMT**	Methylated	13 (30)	7 (37)	20 (32)
	Unmethylated	18 (42)	8 (42)	26 (42)
	Unknown	12 (28)	4 (21)	16 (26)
**Radiation technique**	3D	4 (9)	9 (47)	13 (21)
	IMRT	39 (91)	10 (53)	49 (79)
**GTV initial (cm^3^)**		98 (8–283)	27 (6–137)	
**GTV boost (cm^3^)**		31 (7–165)		
**PTV initial (cm^3^)**		459 (121–1,049)	238 (85–505)	
**PTV boost (cm^3^)**		181 (78–410)		
**Concurrent TMZ**	Yes	43 (100)	15 (79)	58 (94)
	No	0 (0)	4 (21)	4 (6)
**Concurrent bevacizumab**	Yes	13 (30)	7 (37)	20 (32)
	No	30 (70)	12 (63)	42 (68)
**Completed RT**	Yes	37 (86)	17 (90)	54 (87)
	No	6 (14)	2 (10)	8 (13)

KPS, Karnofsky Performance Status; MGMT, O^6^-methylguanine DNA methyltransferase; GTV, gross target volume; PTV, planning target volume; TMZ, temozolomide; RT, radiation therapy.

Data show number (%) or median (range).

**Figure 1:**
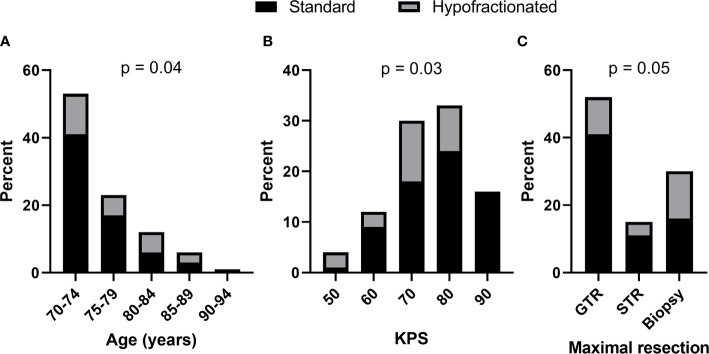
Increased age and decreased KPS were significantly associated with hypofractionated (gray) *vs.* standard (black) adjuvant radiation therapy. Bars show percent of total patients categorized by **(A)** age, **(B)** KPS, and **(C)** extent of maximal resection. Cochran-Armitage test for trend p values shown.

Forty-three (69%) patients received standard RT while 19 (31%) of patients received hypofractionated RT. As shown in **Figure 1**, increased age (p = 0.04, Cochran Armitage test for trend) and decreased KPS (p = 0.03, Cochran Armitage test for trend) were both significantly associated with receipt of hypofractionated RT rather than standard RT. Patients who underwent biopsy only compared to gross or subtotal resection appeared to receive hypofractionated RT more frequently as well, but the association was not significant. RT regimen was not associated with MGMT methylation status or the volume of enhancing tumor, as approximated by the GTV size.

During RT, 13 patients had unscheduled interruptions, and RT was ultimately discontinued early in six receiving standard RT and two receiving hypofractionated RT. Patients who stopped RT early had a median age of 78 (range 71–85), median pre-RT KPS of 80 (range 50–90), and received a median of 66% (range 3–94%) of the prescribed dose. The most common reason for discontinuation was worsening symptoms prompting transition to hospice. Within this small sample, RT discontinuation was not significantly associated with age, pre-RT KPS, extent of maximal resection, RT dose-fractionation, or size of treatment volumes. Following RT, 41 (66%) patients received adjuvant temozolomide for a median of five cycles (range 1–12), and there was no significant association between receipt of adjuvant temozolomide and RT regimen in this series.

**Figure 2:**
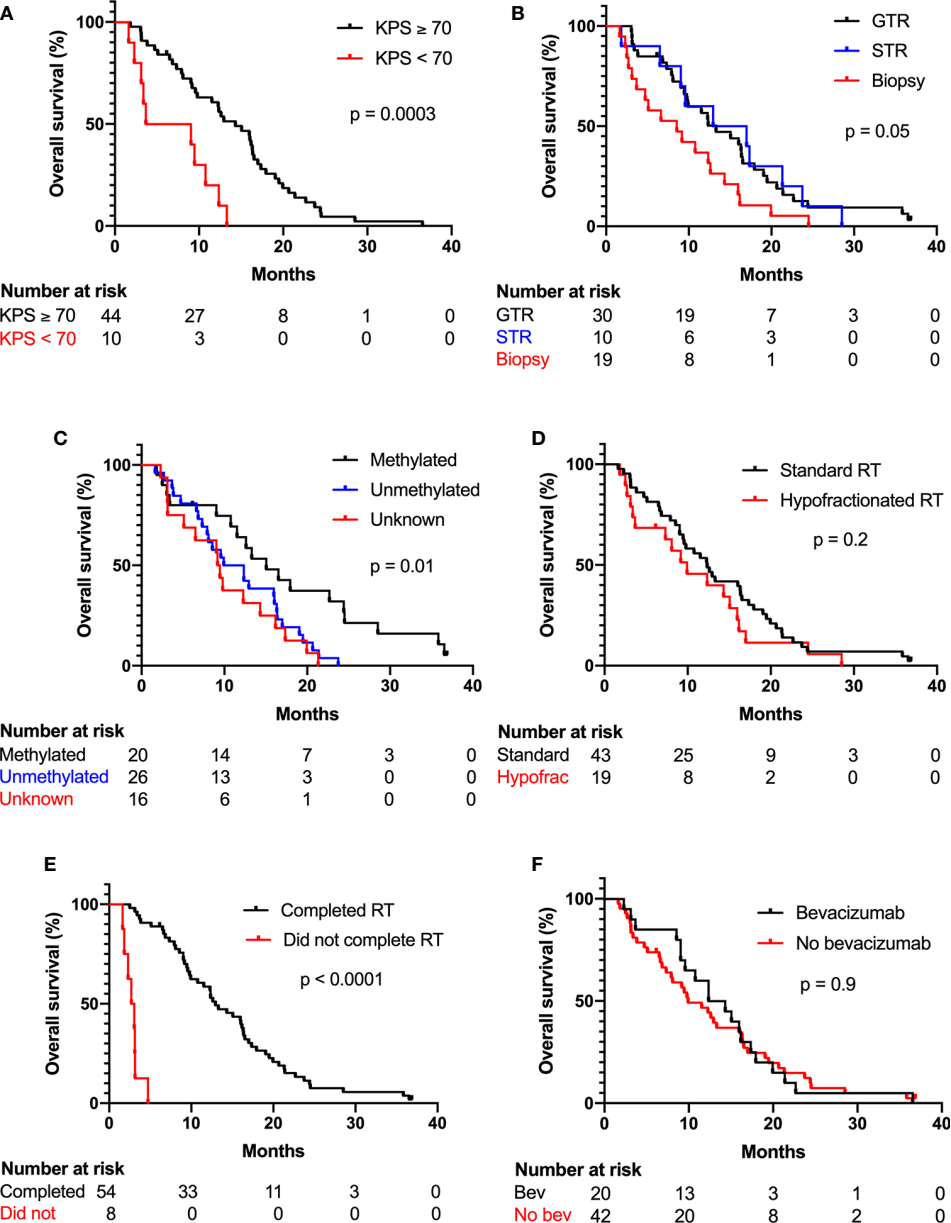
KPS, MGMT methylation status, and RT completion were significantly associated with overall survival. Kaplan-Meier plots show percent overall survival categorized by **(A)** KPS, **(B)** extent of maximal resection, **(C)** methylation status, **(D)** standard or hypofractionated RT, **(E)** completion of RT, and **(F)** receipt of concurrent bevacizumab. Log-rank test p values shown.

Median follow-up time was 37 months, and two patients were alive at last follow-up. Median overall survival was 12.3 months (95% CI 9.0–15.1 months) across all patients. Median overall survival in patients receiving standard RT and hypofractionated RT was 12.4 months (95% CI 9.0–16.4 months) and 9.9 months (95% CI 3.4–15.1 months), respectively. Kaplan-Meier curves of overall survival categorized by KPS, extent of maximal resection, methylation status, hypofractionated *vs.* standard RT, RT completion, and receipt of concurrent bevacizumab are shown in **Figure 2**. On univariate Cox regression analysis, increased age, KPS <70, biopsy *vs.* GTR, unmethylated MGMT *vs.* methylated MGMT, unknown MGMT status *vs.* methylated MGMT, and early RT discontinuation were significantly associated worse survival, as shown in **Table 2**. STR *vs.* biopsy, use of hypofractionated or standard RT, GTV size, and use of concurrent bevacizumab were not significantly associated with survival. On multivariate analysis with the above covariates, only age (HR 1.09, 95% CI 1.01–1.18), KPS <70 (HR 9.29, 95% CI 3.27–26.38), unmethylated MGMT (HR 2.48, 95% CI 1.09–5.64) or unknown MGMT status (HR 3.58, 95% CI 1.31–9.79), and early RT discontinuation (HR 71.76, 95% CI 13.32–386.6) were significantly associated with decreased survival.

**Table 2:** Univariate and multivariate Cox proportional hazards models of overall survival.

Risk factor	Univariate analysis			Multivariate analysis			
	HR	95% CI	p value		HR	95% CI	p value
**Age**	1.08	1.02–1.15	0.01		1.09	1.01–1.18	0.02
**KPS <70 *vs.* KPS ≥70**	3.74	1.74–8.06	<0.001		9.29	3.27–26.38	<0.0001
**Maximal resection**							
GTR *vs.* biopsy	0.51	0.28–0.91	0.02		1.72	0.67–4.41	0.26
STR *vs.* biopsy	0.51	0.23–1.11	0.09		1.01	0.38–2.67	0.98
**MGMT methylation**							
Unmethylated *vs.* methylated	2.37	1.19–4.72	0.01		2.48	1.09–5.64	0.03
Unknown *vs.* methylated	2.95	1.37–6.34	0.01		3.58	1.31–9.79	0.01
**Hypofractionated *vs.* standard RT**	1.39	0.79–2.44	0.25		1.69	0.68–4.18	0.26
**Did not complete *vs.* completed RT**	32.36	9.85–106.3	<0.0001		71.76	13.32–386.6	<0.0001
**GTV size**	1.01	1.00–1.01	0.06		1.00	0.99–1.01	0.59
**Bevacizumab yes *vs.* no**	1.04	0.56–1.64	0.88		0.76	0.38–1.53	0.45

KPS, Karnofsky Performance Status; GTR, gross total resection; STR, subtotal resection; MGMT, O^6^-methylguanine DNA methyltransferase; RT, radiation therapy; GTV, gross target volume.

Data show hazard ratios (HR) and 95% confidence intervals (CI).

## Discussion

Current National Comprehensive Cancer Network (NCCN) guidelines in the United states allow for a range of adjuvant therapies for older GBM patients, including clinical trial, standard RT with temozolomide, hypofractionated RT with temozolomide, temozolomide alone for MGMT methylated patients, or hypofractionated RT alone (10). In the temozolomide era, direct comparisons between standard and hypofractionated RT are limited to retrospective studies, as no randomized data are available. Most retrospective analyses have report similar survival between standard 6-week and hypofractionated 3-week courses of RT (22–27). However, 2 larger series from Italy and California with 129 and 239 patients, respectively, did observe significantly increased survival with standard fractionation (28, 29). A 2019 meta-analysis of 917 patients also detected a significant difference in outcomes, with median OS 13.5 months (95% CI 10.0–16.9) after standard RT and 9.9 months (95% CI 6.5–13.3) after hypofractionated RT both with temozolomide (30).

The present study builds on existing literature and also examines RT details such as GTV size and early RT discontinuation. Similar to prior studies, increased age and poor KPS were significantly associated with selection of hypofractionated rather than standard RT with temozolomide. Median survival following standard and hypofractionated RT was not significantly different at 12.4 and 9.9 months, respectively. Instead, other clinical factors including increased age and poor KPS were associated with decreased survival. Both unmethylated and unknown MGMT status were also associated with poor outcomes, as the latter group likely contained mostly unmethylated patients. Eight (13%) patients discontinued adjuvant RT in the present study due to functional decline, with significantly diminished survival. The majority of these patients had already completed at least half of their RT courses. In this small cohort, no clinical factors were significantly associated with RT discontinuation, and the median pre-RT age was 78 and KPS was 80.

Limitations of the present study as well as other institutional retrospective series include small sample sizes as well as biases in patient and treatment selection. This study includes a highly selected patient population receiving treatment at a tertiary referral center, which may not reflect the patients seen in the community, especially those with limited functional status. This study also included patients ≥70 years old in accordance with NCCN guidelines, however, generally studies of older patients use cutoffs ranging from 65 to 75, making comparison across studies somewhat more challenging (10).

Further investigation into predictors of functional decline may help identify patients where shorter RT courses, palliative care, and other supportive interventions may be more appropriate (31). As noted above, the patients who discontinued RT had similar age and KPS compared to the larger cohort. Thus, in addition to age and KPS, additional measures such as geriatric screening tools and assessments may be helpful to guide selection of adjuvant RT fractionation. For example, the G8 screening tool has been validated in oncology patients >70 years old and more recently in GBM patients ≥65 years old (32, 33). In GBM patients, the G8 score was as stronger predictor of overall survival than age and receipt of radiation or chemotherapy (32). The G8 score also correlated with receipt of standard chemoradiation rather than more radiation alone, chemotherapy alone, or no medical treatment, though all chemoradiation in this study was given per the 6-week Stupp protocol (32). These geriatric screening tools and assessments are also useful for identifying baseline nutrition, mobility, and other functional vulnerabilities that may benefit from early intervention and perhaps even prevent functional decline during RT as well (34).

## Conclusions

In this retrospective single-institution study of 62 GBM patients ≥70 years old who initiated adjuvant RT, median OS was 12.3 months. Age, KPS, MGMT methylation, and RT discontinuation were significantly associated with OS on multivariate analysis, while extent of maximal resection, use of standard or hypofractionated RT, and GTV size were not. Future investigation into factors associated with RT discontinuation and survival may help guide clinical decision-making on RT dose-fractionation and supportive care.

## Data Availability Statement

The raw data supporting the conclusions of this article will be made available by the authors, without undue reservation.

## Ethics Statement

The studies involving human participants were reviewed and approved by Duke University Medical Center Institutional Review Board. Written informed consent for participation was not required for this study in accordance with the national legislation and the institutional requirements.

## Author Contributions

JL: data collection, data analysis, and writing. JK: supervision. FM: formal data analysis. JH: formal data analysis. EL: data curation. AD: supervision. DR: supervision. HF: supervision. DA: supervision. KP: conceptualization, supervision, and project administration. MJ: data collection, writing, conceptualization, and supervision. All authors contributed to the article and approved the submitted version.

## Conflict of Interest

JK reports grants from Varian Medical Systems, others from Clearsight RT Products, LLP, outside the submitted work. AD also receives research funding from Triphase Accelerator Corp., Orbus Therapeutics and Symphogen. AD serves as advisor/speaker/consultant for Istari Oncology and Orbus Therapeutics. AD holds stock/ownership interest with Istari Oncology. AD holds a letter of patent for Oncolytic Polio virus for the treatment of human tumors. HF received compensation for serving as Chief Medical Officer with Istari Oncology. HF holds stock/ownership interest with Istarti Oncology, Diverse Biotech, and Cancer Expert. HF serves as advisor/speaker/consultant for Cancer Expert. HF holds a letter of patent for Oncolytic Polio virus for the treatment of human tumors. KP receives research funding from Agios, BioMimetix, and Novocure. KP serves as advisor/speaker/consultant for Agios and Bayer.

The remaining authors declare that the research was conducted in the absence of any commercial or financial relationships that could be construed as a potential conflict of interest.

## Abbreviations

RT, radiation therapy; GBM, glioblastoma; IRB, institutional review board; KPS, Karnofsky performance status; OS, overall survival; MGMT, O^6^-methylguanine DNA methyltransferase MGMT.
